# Characteristics associated with regular attendance and quality of asthma annual reviews in adults with asthma in England

**DOI:** 10.1038/s41533-026-00514-5

**Published:** 2026-04-28

**Authors:** Hannah Whittaker, Jennifer K. Quint

**Affiliations:** Department of Primary Care and Public Health, School of Public Health, Imperial College London, London, UK

**Keywords:** Diseases, Health care, Medical research, Risk factors

## Abstract

Routinely collected electronic healthcare records from the Clinical Practice Research Datalink Aurum were used to investigate patient characteristics associated with attendance and regular attendance of asthma annual reviews. Females, older age, ex-smokers, White ethnicity, lower socioeconomic deprivation, and comorbidities were associated with higher rates of asthma annual reviews however, females, ex-smokers, and depression were associated with less regularly reviews, indicating the sporadic nature of reviews, despite guidelines.

Asthma is one of the most prevalent chronic conditions in Europe, affecting an estimated 6.7% of the population, with the highest prevalence reported in the United Kingdon (UK)^1,2^. Improved asthma control can significantly reduce exacerbations and hospitalisations, thereby alleviating the burden on healthcare resources. In the UK, asthma annual reviews (AARs) for people with asthma are offered in primary care to review the condition, evaluate asthma control, and support self-management^3^. In England, the Quality and Outcomes Framework (QOF) asthma indicator encourages provision of good quality asthma reviews, and general practices are awarded financial incentives based on the percentage of patients with asthma on the asthma register who have had an AAR in the preceding 12 months^4^. The AAR process typically includes assessment of symptoms and risk, review of inhaled therapy and adherence, inhaler technique review, trigger and smoking assessment, and an updated written asthma action plan where appropriate^3^.

Despite efforts to improve asthma control in patients with asthma, evidence suggests that attendance of AARs is low. In 2005, one third of people with asthma registered at a small number of general practices in England had an AAR^5^. In Wales, 54.4% and 39.8% of adults and children with asthma, respectively, had an AAR between 2021–2023^6^. Recently in Scotland, 84% of patients attended an AAR more than once over a median of 7.9 years^7^.

Given this, it is important to understand which patients are more or less likely to attend AARs to implement optimal interventions in the future. Similarly, there is limited evidence on how AARs are carried out in England and which components of the AAR are performed and recorded during these specialised visits. We aimed to understand which groups of patients with asthma have more frequent, regular, and better recorded AARs using routinely collected healthcare records in England.

A total of 244,576 patients with asthma were included in the study, with a median follow-up of 4.0 years (IQR 1.9–7.1). Of these, 78,746 (32.2%) patients had at least one AAR over follow-up. In those who had at least one annual review over follow-up, the median annual rate was 0.48 per year (IQR 0.26-0.79). Out of the study population, 145,269 (59.4%) were female, mean age was 50.2 years (SD 18.1), 204,381 (85.1%) had White ethnicity, mean BMI was 27.3 kg/m² (SD 5.0), 60,007 (24.6%) were current smokers, 126,711 (51.9%) were ex-smokers, 14,835 (6.1%) had depression, and there was an even distribution across IMD quintiles.

Crude estimates are reported in supplementary table 1. Higher rates of AARs were seen in females, older patients, ex-smokers, patients with White ethnicity, patients living in areas with lower socioeconomic deprivation, and patients with higher BMI (Fig. 1). These results suggest that there may be groups of people who are less likely to attend AARs for various reasons. Reasons for non-attendance may include differences in healthcare-seeking behaviours, access to general practice, lack of awareness of the importance of AARs, and milder disease^8,9^. Whilst we adjusted for baseline measures, including prior exacerbations and consultation frequency, asthma severity may change over the course of follow-up.

**Fig. 1:**
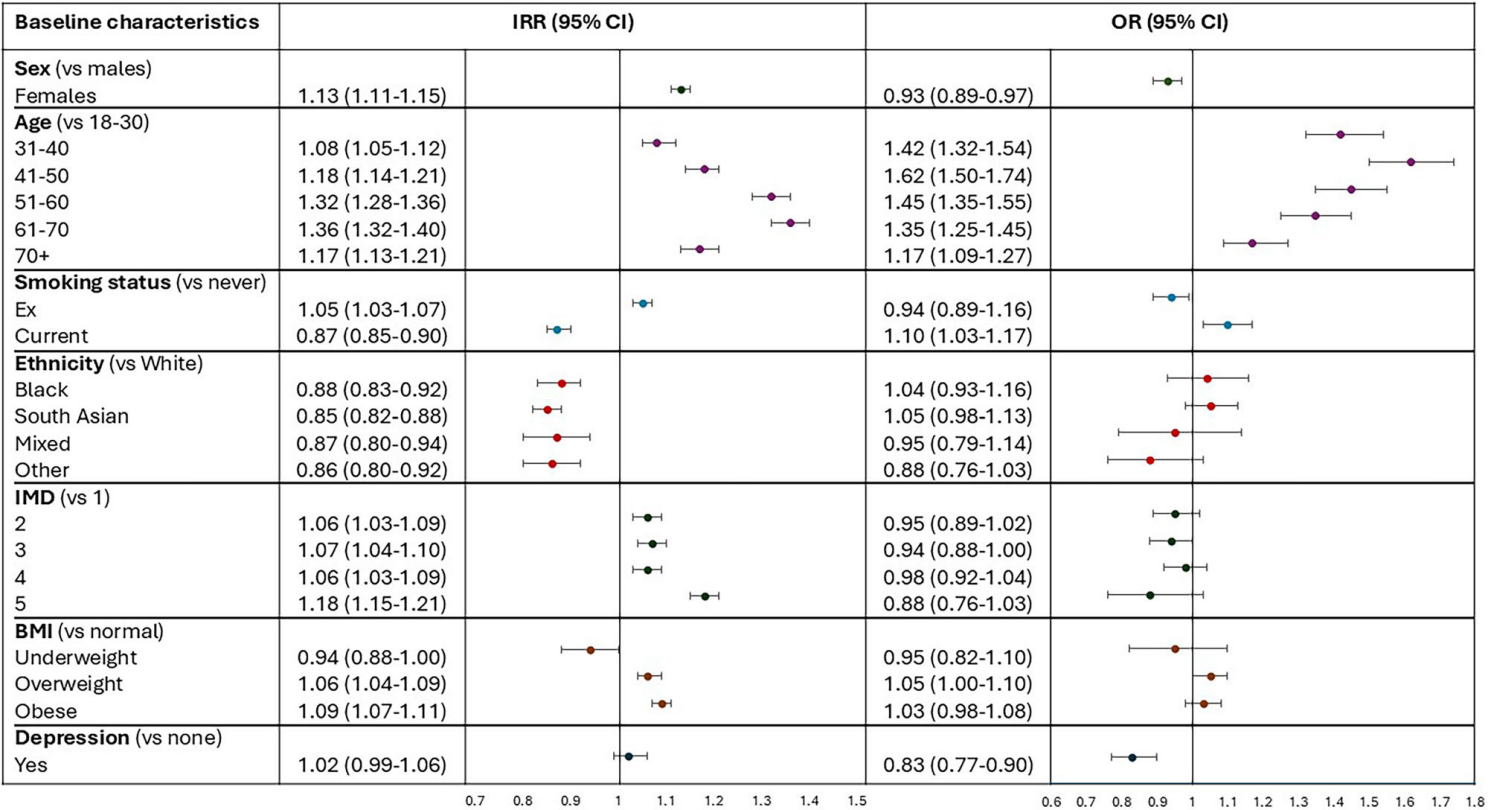
Association between patient characteristics and asthma annual reviews. Estimates report IRR for the association between baseline characteristics and rate of asthma annual reviews over follow-up and OR for the association between baseline characteristics and odds of having regular asthma reviews in those with at least one asthma annual review. IRR (incidence rate ratio), CI (confidence interval), OR (odds ratio), IMD (index of multiple deprivation), BMI (body mass index). IMD 1 is the least deprived and IMD 5 the most deprive quintile.

Among those with at least one AAR over follow-up, the odds of having regular AARs were higher in males, those aged 41–50 years, current smokers, and patients who did not have depression (Fig. 1). Alongside the previous findings, these results suggest that while some groups of patients may be more likely to have AARs, some may be less likely to have regular reviews. For example, females had higher rates of asthma reviews, but among those who attended a review, females were less likely to have regular reviews compared with males. This was also seen for ex-smokers and those diagnosed with depression. We reported nurse-related AARs only, as these are intended to be conducted at a general practice during a longer consultation to review a patient’s asthma. In practice, asthma assessment may also occur opportunistically in other consultations, for example if a general practitioner receives a prompt that the patient has not had an asthma review within the year. It is possible that patients who are more likely to seek healthcare regularly have their reviews conducted when they see healthcare practitioners for other reasons. For example, it is known that females seek healthcare more than males^10^. On the other hand, people who have other comorbidities, such as depression, likely attend their general practice for other conditions, which might be why they also have less regular asthma reviews^11^. Sensitivity analyses using AARs recorded by any healthcare practitioner yielded similar results.

Lastly, we found that the median proportion of AARs with inhaler technique and asthma management plan recorded was 60% (IQR 0–100) and 20% (IQR 0–75), respectively. It is possible that inhaler technique or an asthma management plan was assessed but not recorded. Having a record of a personalised management plan during an asthma review is reported in QOF and is therefore surprising that not all reviews had this recorded in our study^4^. It is possible that these components are recoded as free text, which would explain these results. On the other hand, a record of an inhaler technique assessment is not required for QOF, which may be why a median of 60% were recorded within an asthma review. More complete and consistent recording of key AAR components would improve the ability to assess delivery of recommended care.

Overall, our findings highlight the disparities in attending AARs in England and warrant further investigation into the knock-on effects of not attending AARs to help reduce the overall burden of asthma.

## Methods

### Data source

Primary care data from the Clinical Practice Research Datalink (CPRD) Aurum were used. CPRD Aurum contains deidentified data on approximately 20% of patients registered at general practices in England and Wales and is generalisable to the English population in terms of age, sex, geographic spread and socioeconomic deprivation^12^. Our study population included patients with a primary care diagnosis of asthma aged 18 years or older registered at a general practice between 2010 and 2019. Index date was the patient’s asthma diagnosis date, and follow-up ended on 31/12/2019 or earlier if patients died or left their general practice.

AARs were defined using QOF-specific SNOMED CT codes for asthma reviews conducted by a general practice nurse over follow-up. Regular AAR attendance was defined as having an asthma review at least every 15 months over the patient’s entire follow-up (i.e., no gap greater than 15 months between consecutive reviews) following guidelines^4^. Components of the AAR were also defined and included inhaler technique and asthma management plan over follow-up. Baseline exposure variables included age at index date, sex, closest smoking status prior to index date, ethnicity, socioeconomic status, body mass index (BMI), and depression defined within five years of the patient’s index date.

### Statistical analyses

Multivariable negative binomial regression and logistic regression investigated the association between patient characteristics and rates of AARs and odds of having regular AARs, respectively. Negative binomial models used person-years as the offset. Models were additionally adjusted for history of general practice visits and baseline exacerbations in the year prior to index date as a proxy of disease severity. Secondly, the proportion of AARs with recorded inhaler technique and asthma management plan was described over follow-up using descriptive statistics (medians and interquartile ranges).

### Ethical approval

Aprotocol for this research was approved by the independent scientific advisory committee (ISAC) for the UK Medicines and Healthcare products Regulatory Agency (MHRA) Database Research (protocol No 23_003115), and the approved protocol was made available to the reviewers during peer review. This work is based on data from the CPRD obtained under license from the MHRA. Informed consent was not required due to the use of de-identified patient data. The interpretation and conclusions contained in this study are those of the authors alone.

## Supplementary information

Supplementary material

## Data Availability

Data may be obtained from a third party and are not publicly available. Data sets generated and/or analysed in this study are not publicly available, however, data are available on request from the CPRD. Their provision requires the purchase of a license and this license does not permit the authors to make them publicly available to all. This work used data from the version collected in July 2023 and has clearly specified the data selected in the Methods section. To allow identical data to be obtained by others, via the purchase of a license, the code lists will be provided upon request. Licenses are available from the CPRD (http://www.cprd.com): The Clinical Practice Research Datalink Group, The Medicines and Healthcare products Regulatory Agency, 10 South Colonnade, Canary Wharf, London E14 4PU.

## References

[1] Mukherjee, M. et al. The epidemiology, healthcare and societal burden and costs of asthma in the UK and its member nations: analyses of standalone and linked national databases. *BMC Med*. **14**, 113 (2016). 27568881 10.1186/s12916-016-0657-8 PMC5002970

[2] Khan, A. H. et al. Prevalence and burden of asthma in five European countries: a retrospective cross-sectional study. *BMJ Open* **15**, e085175 (2025). 40409961 10.1136/bmjopen-2024-085175 PMC12104913

[3] National Institute for Health and Care Excellence (NICE). Asthma: annual review. (2019). [accessed 06/02/26].

[4] Quality and Outcomes Framework (QOF). Business Rules for Quality and Outcomes Framework (QOF) 2025: Asthma. 2025 [accessed 06/02/26].

[5] Pinnock, H. et al. Cost-effectiveness of telephone or surgery asthma reviews: economic analysis of a randomised controlled trial. *Br J Gen Pract* **55**, 119–124 (2005). 15720933 PMC1463186

[6] National Respiratory Audit Programme (NRAP). Wales primary care audit report. (2024) [accessed 06/02/26].

[7] Tibble, H. et al. Prevalence and predictors of annual asthma reviews in Scottish primary care data. *BJGP Open* 0062 (2024). 10.3399/BJGPO.2024.0062 PMC12138009 39357905

[8] Mault, S., McDonough, B., Currie, P. & Burhan, H. P278 Reasons proffered for non-attendance at a difficult asthma clinic. *Thorax* **67**, A187 (2012).

[9] Primary Care Respiratory Society (PCRS). Why I hate asthma reviews. (2019) [accessed 06/02/26].

[10] Holm, A. et al. Do men avoid seeking medical advice? A register-based analysis of gender-specific changes in primary healthcare use after first hospitalisation at ages 60+ in Denmark. *J Epidemiol Community Health* **0**, 1–7 (2020). 10.1136/jech-2019-213435 PMC7337231 32303595

[11] Payne, R. A. et al. The effect of physical multimorbidity, mental health conditions and socioeconomic deprivation on unplanned admissions to hospital: a retrospective cohort study. *CMAJ* **185**, E221–E228 (2013). 23422444 10.1503/cmaj.121349 PMC3602270

[12] Wolf, A. et al. Data resource profile: Clinical Practice Research Datalink (CPRD) Aurum. *Int J Epidemiol* **48**, 1740–1740g (2019). 30859197 10.1093/ije/dyz034 PMC6929522

