# Supplementary material for: Characteristics associated with regular attendance and quality of asthma annual reviews in adults with asthma in England
Source: NPJ Prim Care Respir Med. 2026 Apr 28;36:44. doi: 10.1038/s41533-026-00514-5 (PMC13328552; doi:10.1038/s41533-026-00514-5)
Supplement: Supplementary file 1 — Supplementary material [file 41533_2026_514_MOESM1_ESM.docx]

**Characteristics associated with regular attendance and quality of asthma annual reviews in adults with asthma in England**

Supplementary material

Contents

[Table 1: Crude IRR and OR for the association between patient characteristics and asthma annual reviews. 2](#_Toc222909834)

# Table 1: Crude IRR and OR for the association between patient characteristics and asthma annual reviews.

| **Patient characteristic** | **Crude IRR (95% CI)** | **Crude OR (95% CI)** |
| --- | --- | --- |
| **Sex** Males Females | Ref 1.15 (1.13-1.17) | Ref  0.90 (0.86-0.93) |
| **Age** 18-30 31-40 41-50 51-60 61-70 70+ | Ref 1.13 (1.09-1.16) 1.29 (1.25-1.32) 1.48 (1.43-1.52) 1.56 (1.51-1.60) 1.37 (1.33-1.42) | Ref 1.37 (1.28-1.47) 1.59 (1.49-1.70) 1.40 (1.32-1.50) 1.31 (1.23-1.40) 1.09 (1.02-1.17) |
| **Smoking status** Never smoker Ex-smoker Current smokers | Ref 1.17 (1.14-1.19) 0.96 (0.93-0.98) | Ref 0.94 (0.89-0.98) 1.08 (1.02-1.14) |
| **Ethnicity** White Black South Asian Mixed  Other | Ref 0.88 (0.84-0.93) 0.84 (0.80-0.86) 0.81 (0.75-0.87) 0.82 (0.77-0.88) | Ref 1.06 (0.95-1.17) 1.07 (1.00-1.15) 0.97 (0.82-1.16) 0.92 (0.79-1.07) |
| **IMD** 1 (least deprived) 2 3 4 5 (most deprived) | Ref 1.05 (1.03-1.08) 1.05 (1.03-1.08) 1.02 (0.99-1.05) 1.13 (1.10-1.16) | Ref 0.95 (0.89-1.01) 0.95 (0.89-1.01) 0.99 (0.93-1.05) 0.94 (0.89-1.00) |
| **BMI** Normal  Underweight  Overweight Obese | Ref 0.90 (0.84-0.96) 1.11 (1.09-1.14) 1.19 (1.17-1.22) | Ref 0.89 (0.77-1.03) 1.07 (1.02-1.12) 1.02 (0.97-1.07) |
| **Depression** No  Yes | Yes 1.08 (1.04-1.12) | Ref 0.81 (0.75-0.87) |

Legend: Number of patients included in crude IRR analysis: 244,576 for all models, except for 244,225 for smoking status, 240,103 for ethnicity, 244,255 for IMD, and 220,349 for BMI. Number of patients included in adjusted IRR analysis: 216,839. Number of patients included in crude OR analysis: 78,746, expect for 78,742 for smoking status, 77,866 for ethnicity, 78,708 for IMD, and 73,436 for BMI. Number of patients included in adjusted OR analysis: 72,653.
